# Supplementary material for: The Scaly-foot Snail genome and implications for the origins of biomineralised armour
Source: Nat Commun. 2020 Apr 8;11:1657. doi: 10.1038/s41467-020-15522-3 (PMC7142155; doi:10.1038/s41467-020-15522-3)
Supplement: Supplementary file 1 — Supplementary Information [file 41467_2020_15522_MOESM1_ESM.pdf]

Supplementary Information for:

The scaly-foot snail genome and implications for the origins of biomineralised armour

Nature Communications

Jin Sun, Chong Chen, Norio Miyamoto, Runsheng Li, Julia D. Sigwart, Ting Xu, Yanan Sun, Wai Chuen Wong, Jack C.H. Ip, Weipeng Zhang, Yi Lan, Dass Bissessur, Tomo-o Watsuji, Hiromi Kayama Watanabe, Yoshihiro Takaki, Kazuho Ikeo, Nobuyuki Fujii, Kazutoshi Yoshitake, Jian-Wen Qiu, Ken Takai, & Pei-Yuan Qian

Correspondence should be addressed to P-YQ (boqianpy@ust.hk) or KT (kent@jamstec.go.jp)

Table of Contents

Supplementary Figures

1. Properties of the *Chrysomallon squamiferum* genome …..……………………………………1

2. Enriched Gene Ontology (GO) terms for the expanded gene families ……………………… 2

3. Chromosome-scale macro-synteny comparison ……………………………………………... 3

4.Shuffling of Hox-like genes in comparison to other lophotrochozoans …………………….. 4

5. Expansion of the DMBT1 gene family …………………...……………………………… 5

6. Characterisation of chitin synthase ……………..………………………………………… 6

7. Characterisation of the metal tolerance protein (MTP) ………………………………………7

Supplementary Tables

1. Summary statistics of the assembled *Chrysomallon squamiferum* genome …….………… 8

2. Characteristics of high quality lophotrochozoan genomes ………………………………… 9

3. Number of low complexity proteins in different tissues types ……………………………….10

4. Transcription factors involved in hard structure formation in Lophotrochozoa …….………11

Supplementary Notes

1. Background information on the scaly-foot snail ……………………………………….…… 12

2. Genome sequencing, assembly, and annotation ……………….…………………………..… 14

3. Chromosomal distribution of genes ……………………………………….……………… 20

4. Transcriptome Gene Ontology enrichment analyses ………………………………………… 21

5. Real-time PCR validation ………………………………..…..…………………………… 23

6. Gene family and phylogenetic analyses ……………………………………………………... 25

Supplementary References (for all information in this document) ………………………… 26

The source data underlying Supplementary Figures 1, 2, 3, 6b, 7c, 8, 9, 10, 11, 12, and 13 are provided as a **Source Data** file.

Supplementary Figures

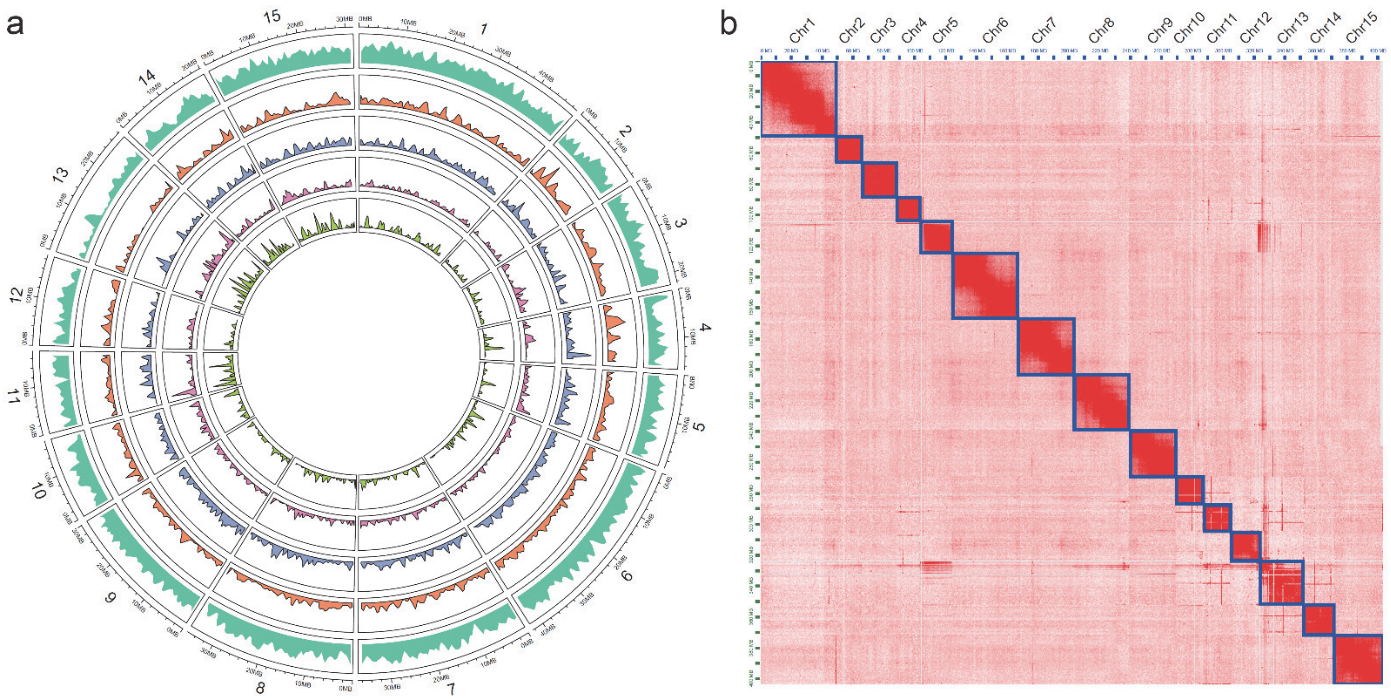

Supplementary Figure 1. Properties of the *Chrysomallon squamiferum* genome. a, Circos plot showing the gene density and the distribution of tissue highly expressed, genes across the whole genome of the Scaly-foot Snail. From outside to inside, each circle represents: the gene density in each pseudo-chromosome, the genes that were highly expressed in the gill, the oesophageal gland, the shell-secreting mantle, and scales. The sliding window size is 10kb. **b**, The 15 pseudo-chromosomes in the assembled Scaly-foot Snail genome shown by Hi-C linkage density maps. The color value reflects the log-scaled number of valid read pairs within 100kb window size. Source data are provided as a Source Data file.

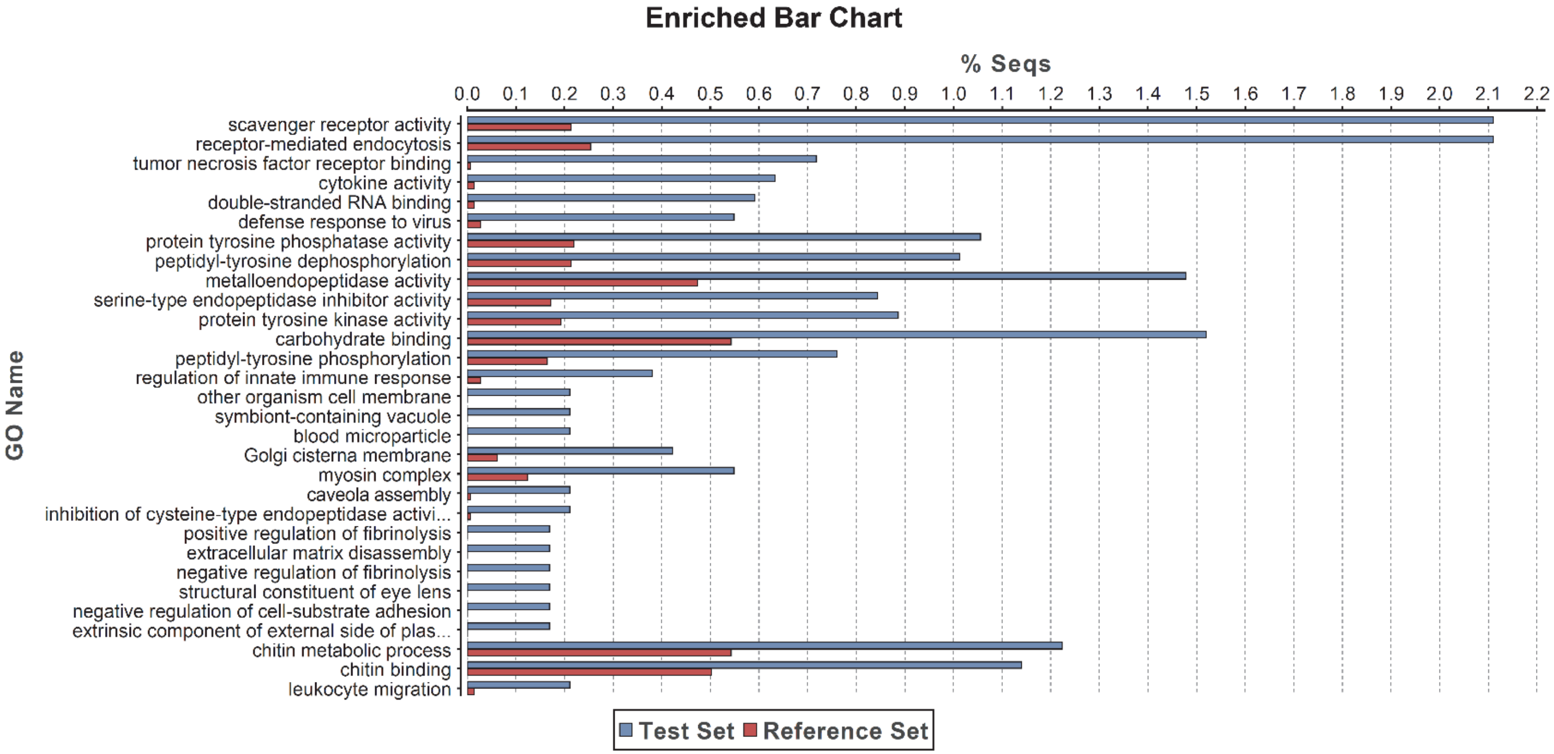

Supplementary Figure 2. Enriched Gene Ontology (GO) terms of the expanded gene families in *Chrysomallon squamiferum*. Blue and red bars indicate the percentage of genes in the expanded gene families and all genes in each GO term, respectively. Analysis was performed by BLAST2GO. Source data are provided as a Source Data file.

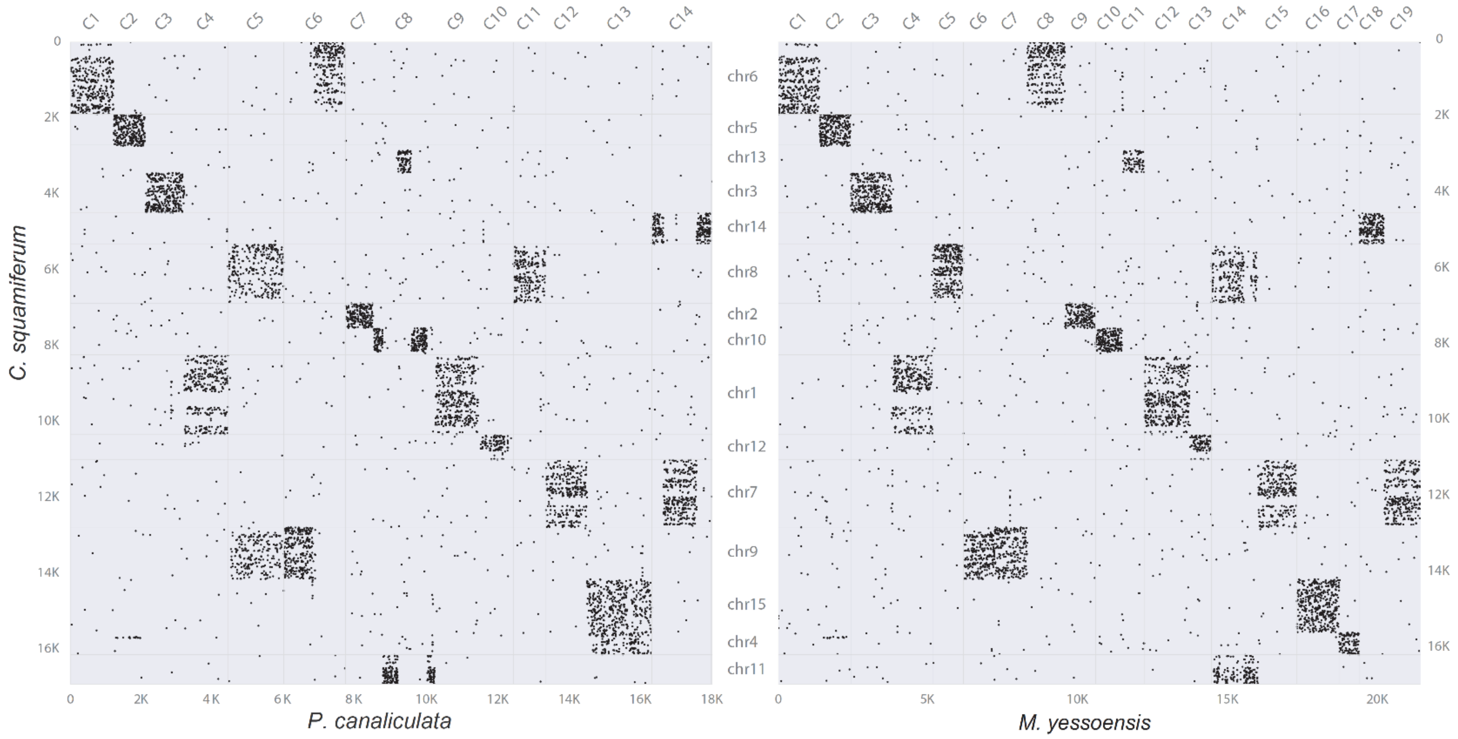

Supplementary Figure 3. Chromosome-scale macro-synteny comparison. Dot-plots showing chromosome-scale macro-synteny comparison between the Scaly-foot Snail and the apple snail *Pomacea canaliculata* (left) and between the Scaly-foot Snail and the scallop *Mizuhopecten yessoensis* (right). Each dot represents the mutual protein best match between each pair of species. In total, 8544 gene pairs were found between *Chrysomallon squamiferum* and *P. canaliculata* and 7845 gene pairs between *C. squamiferum* and *M. yessoensis*. The genes of each species were sorted according to their genomic positions; and the accumulative number of genes in each species is shown. Source data are provided as a Source Data file.

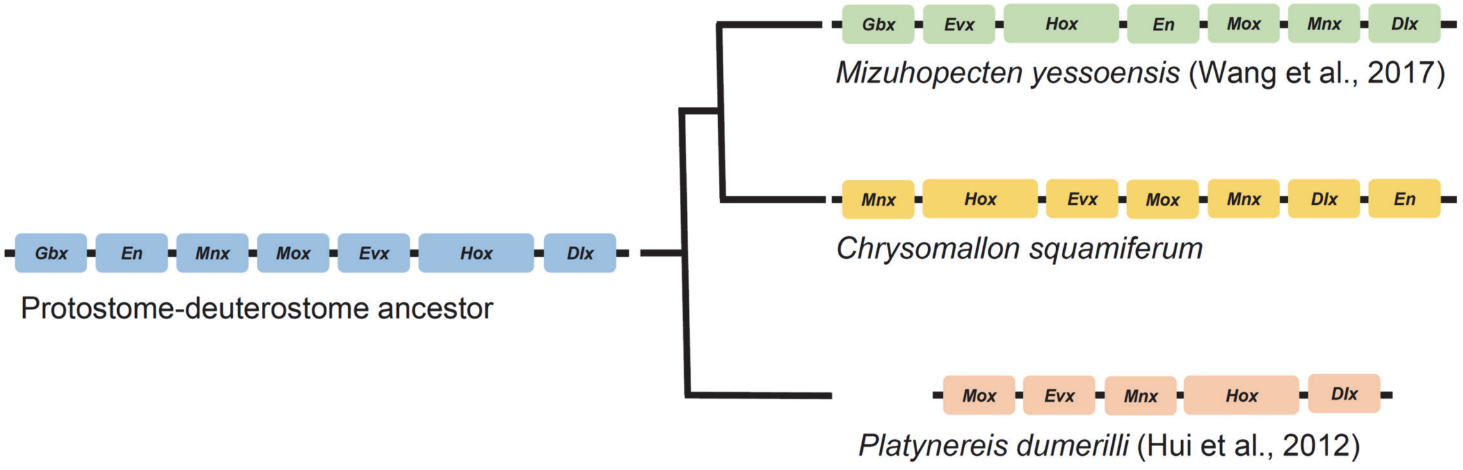

Supplementary Figure 4. Shuffling of Hox-like genes in *Chrysomallon squamiferum*, in relation to other lophotrochozoans. Hox-like gene positions within the Hox chromosome are shown in the deduced proteome-deuterostome ancestor[1]: the Scaly-foot Snail *Chrysomallon squamiferum*, the scallop *Mizuhopecten yessoensis*[2], and the annelid worm *Platynereis dumerilii*[1] (unsequenced part shown as empty).

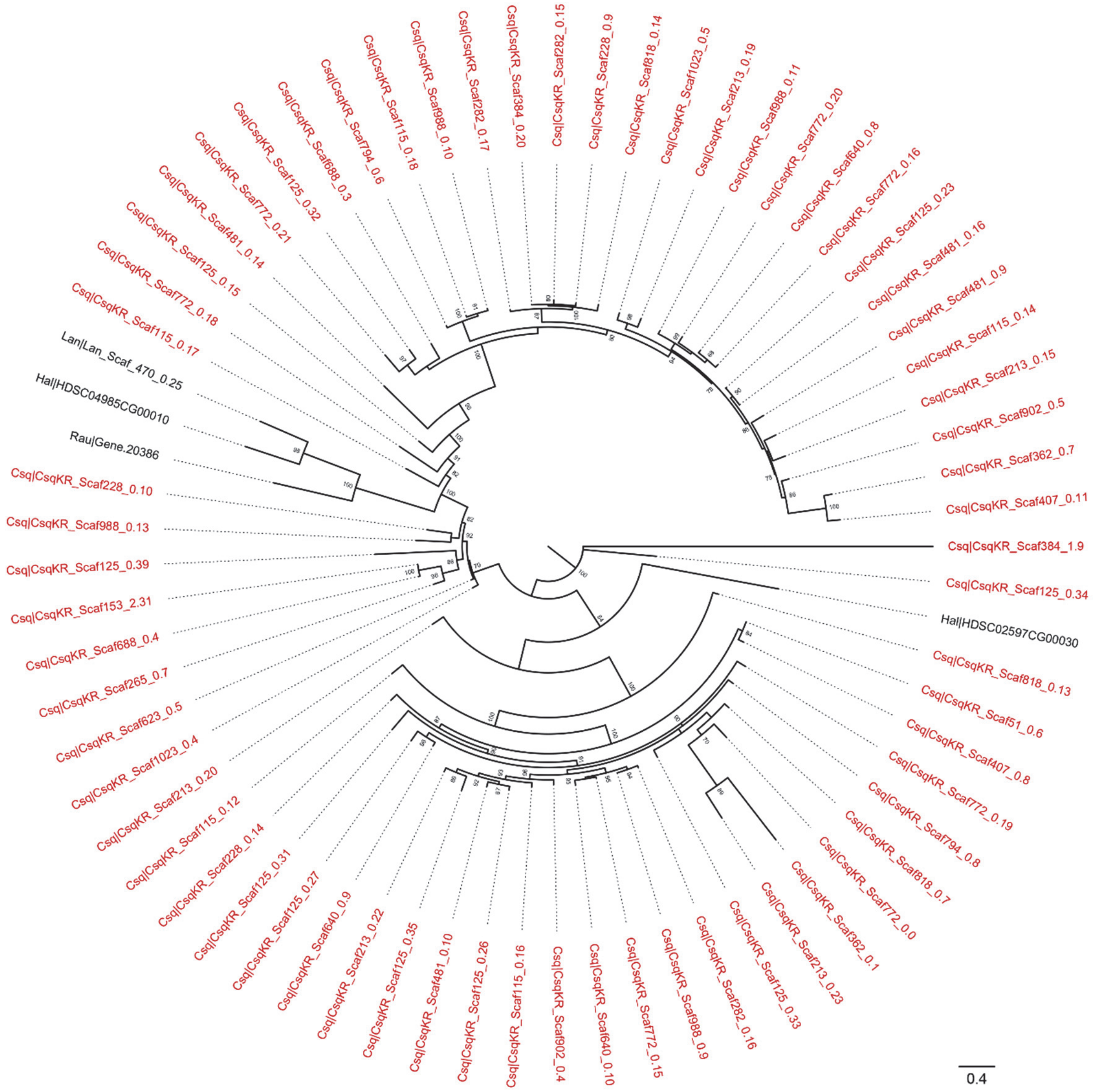

Supplementary Figure 5. Expansion of the DMBT1 gene family in *Chrysomallon squamiferum.* Unrooted genealogy of DMBT1 genes in *Chrysomallon squamiferum* (Csq, red), *Haliotis discus hannai*, (Hdi, black), *Lanistes nyassanus* (Lan, black) and *Radix auricularia* (Rau, black).

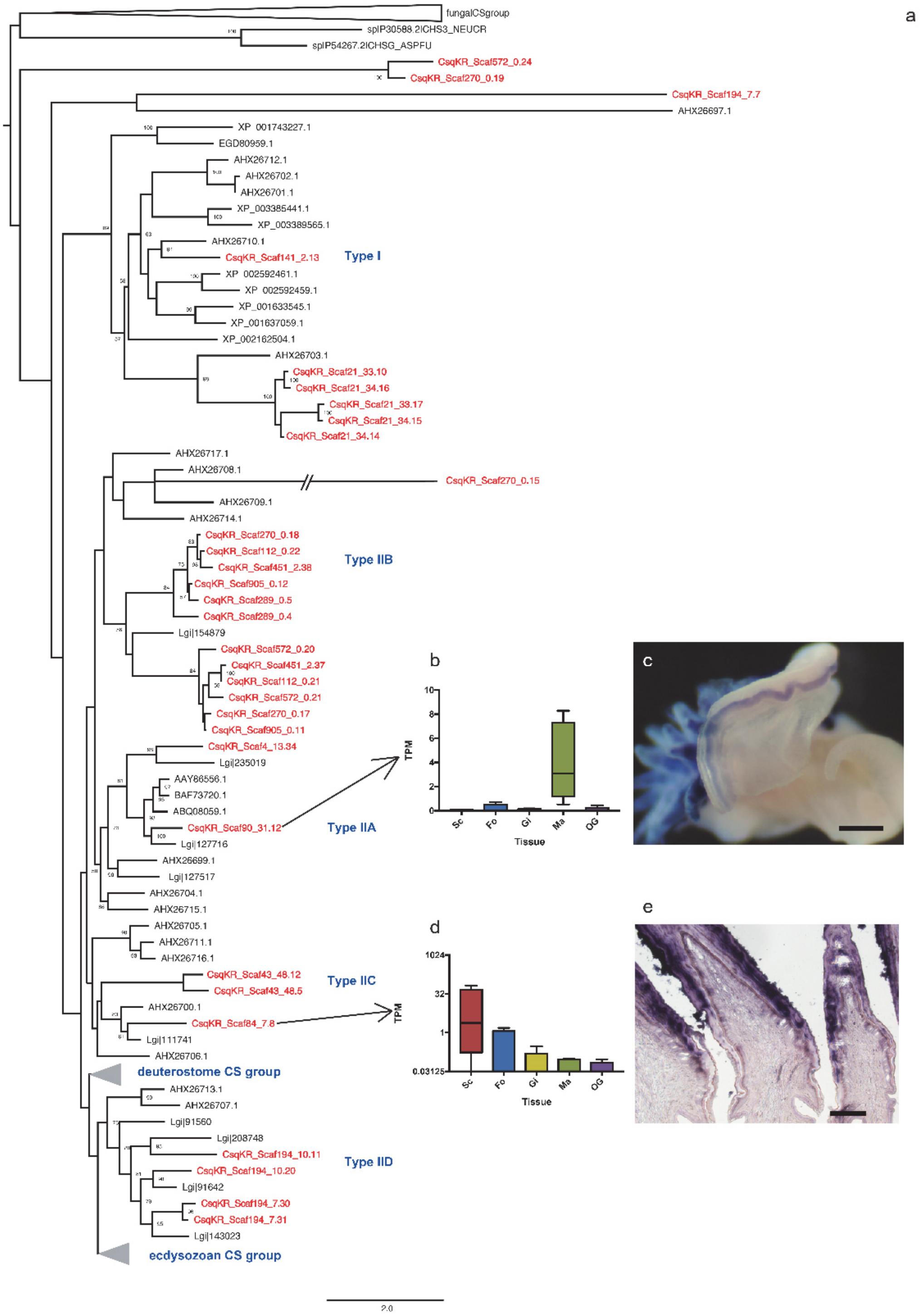

Supplementary Figure 6. Characterisation of chitin synthase in *Chrysomallon squamiferum*. **a**, Phylogenetic analysis of chitin synthase (CS) gene with names in red, indicating genes from the Scaly-foot Snail. **b**, Boxplot showing the expression level of CS IIA in different tissues (n = 5 biologically independent animals). Source data are provided as a Source Data file. **c**, *In situ* hybridization showing expression of CS IIA in the mantle. **d**, Boxplot showing the expression level of CS IIC in different tissues (n = 5 biologically independent animals). **e**, *In situ* hybridization showing expression CS IIC in the scales. Within the Boxplot, the centre line refers to the median, and the boxplot depicts the first to the third quartile, with the whiskers indicating maximum and minimum expression levels. Abbreviations: Fo, Foot; Gi, Gill; Ma, Mantle; OG, Oesophageal gland; Sc, Scale. Scale bars = 500 µm for mantle and 100 µm for scales.

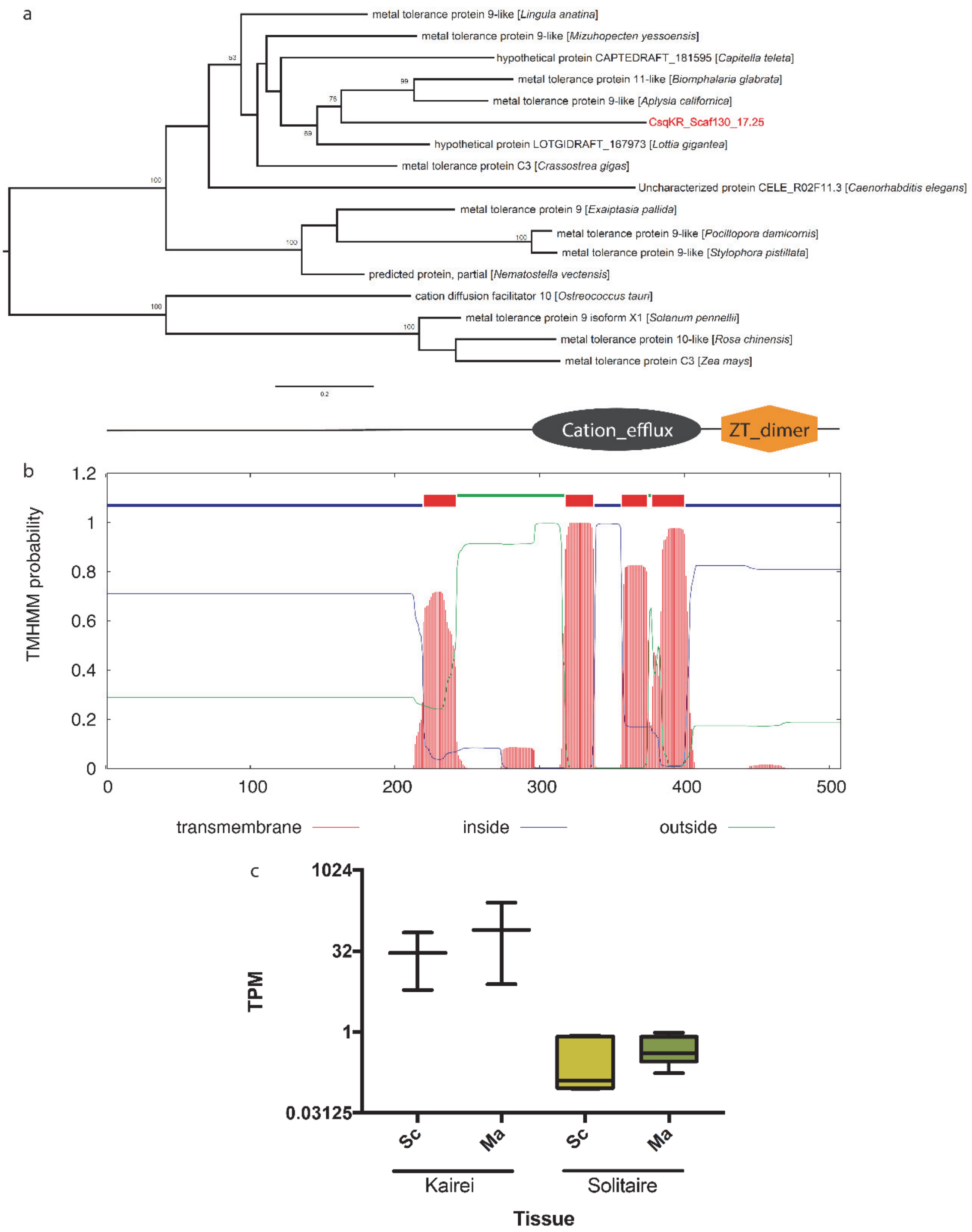

Supplementary Figure 7. Chracterisation of the metal tolerance protein (MTP) in *Chrysomallon squamiferum.* **a**, phylogenetic analysis of the MTP, with the name in red indicating the Scaly-foot snail MTP. **b**, functional domain and TMHMM analysis of the MTP, indicating the existence of two domains: one cation efflux domain located at the transmembrane region, and another ZT dimer located at the extracelluar region. **c**, the expression level of the MTP in the two hard-part secreting tissues of *C. squamiferum*, scale (Sc) and mantle (Ma), showing comparison between three individuals from the iron-rich Kairei field (*n* = 3 biologically independent animals, i.e. E02B1, B2, E02B2) and five individuals from the iron-deprived Solitaire field (*n* = 5 biologically independent animals, i.e. IW1, IW2, IW3, W2, and W7). Within the Boxplot, the centre line refers to the median, and the boxplot depicts the first to the third quartile, with the whiskers indicate maximum and minimum expression levels. Source data are provided as a Source Data file.

Supplementary Tables

Supplementary Table 1. Summary statistics of the assembled *Chrysomallon squamiferum* genome.

Assembly feature	Statistics
Estimated genome size (by 17-mer analysis)	444.4 Mb
Number of scaffolds	22*
Total assembly size (bp)	404,615,235
Longest scaffold (bp)	49,219,174
N50 scaffold length (bp)	30,197,626
L50 scaffold count	6
N50 contig length (bp)	1,883,489
L50 contig count	54
GC content	29.82%
Gene number predicted	16,917
Mean exon length (bp)	233
Mean intron length (bp)	1109
Mean exon count per gene	7.2

*15 pseudo-chromosome linkage groups + 7 small unanchored contigs

Supplementary Table 2. Characteristics of high quality lophotrochozoan genomes.

Data for *Chrysomallon squamiferum*, *Pomacea canaliculata*[3], *Radix auricularia*[4], *Aplysia californica* (GenBank No. GCA_000002075), *Biomphalaria glabrata*[5], *Lottia gigantea*[6], *Elysia chlorotica*[7], *Haliotis discus hannai*[8], *Achatina fulica*[9], *Crassostrea virginica* (GenBank No. GCA_002022765.4), *Crassostrea gigas*[10], *Mizuhopecten yessoensis*[2], *Azumapecten farreri*[11], *Sinonovacula constricta*[12], *Ruditapes philippinarum*[13], *Bathymodiolus platifrons*[14], *Modiolus philippinarum*[14], *Pinctada fucata*[15], *Architeuthis dux*[16], *Octopus bimaculoides*[17], *Euprymna scolopes*[18], *Lingula anatina*[19], *Notospermus geniculatus*[20], *Phoronis australis*[20], *Capitella teleta*[6], *Lamellibrachia luymesi*[21], and *Helobdella robusta*[6]. BUSCO, Benchmarking Universal Single-Copy Orthologs. The column shows the percentage of complete single-copy BUSCOs.

Group	Species	Assembled length	No. of scaffolds	Longest scaffold	Unknown sequences	Scaffold N50	Contig N50	%BUSCOs
Gastropoda	*Chrysomallon squamiferum*	405 Mb	22	49.2 Mb	0.00005	30.2 Mb	1.88Mb	96.60%
Gastropoda	*Pomacea canaliculata*	440 Mb	24	45.4 Mb	0.0002	31.5 Mb	1.10 Mb	95.80%
Gastropoda	*Radix auricularia*	910 Mb	4823	2.97 Mb	0.0642	579 Kb	26.7 Kb	93.10%
Gastropoda	*Aplysia californica*	927 Mb	4332	610 Kb	0.2044	918 Kb	9.6 Kb	92.40%
Gastropoda	*Biomphalaria glabrata*	916 Mb	331,401	2.18 Mb	0.019	48.1 Kb	16.6 Kb	88.30%
Gastropoda	*Lottia gigantea*	360 Mb	4475	9.39 Mb	0.1686	1.87 Mb	96.0 Kb	95.80%
Gastropoda	*Elysia chlorotica*	558 Mb	9989	2.35 Mb	0.03	442 Kb	32.2Kb	93.10%
Gastropoda	*Haliotis discus hannai*	1865 Mb	80,032	2.21 Mb	0.0625	200 Kb	41.0 Kb	91.50%
Gastropoda	*Achatina fulica*	1856 Mb	1500	116.6 Mb	0.0019	59.6Mb	721.0Kb	86.50%
Bivalvia	*Crassostrea virginica*	684.7 Mb	10	104.2 Mb	0.0001	75.9 Mb	1.97 Mb	94.60%
Bivalvia	*Crassostrea gigas*	558 Mb	7659	1.96 Mb	0.1181	402 Kb	32.6 Kb	95.00%
Bivalvia	*Azumapecten farreri*	910 Mb	388,151	6.57 Mb	0.0155	669 Kb	43.7 Kb	94.10%
Bivalvia	*Sinonovacula constricta*	1221 Mb	362	97.4 Mb	0.0067	65.9 Mb	978 Kb	92.30%
Bivalvia	*Ruditapes philippinarum*	1123 Mb	26,963	59.1 Mb	0.054	46.3 Mb	31.3 Kb	92.20%
Bivalvia	*Bathymodiolus platifrons*	1658 Mb	65,662	2.79 Mb	0.1177	343 Kb	13.2 Kb	93.50%
Bivalvia	*Pinctada fucata*	815 Mb	29,306	1.26 Mb	0.0671	167 Kb	21.9 Kb	92.00%
Bivalvia	*Mizuhopecten yessoensis*	988 Mb	82,659	7.50 Mb	0.081	804 Kb	66.8 Kb	93.10%
Bivalvia	*Modiolus philippinarum*	2630 Mb	74,573	715 Kb	0.048	100 Kb	19.7 Kb	89.50%
Cephalopoda	*Euprymna scolopes*	5710 Mb	50,192	29.7 Mb	0.33	3.70 Mb	98.0 Kb	96.90%
Cephalopoda	*Architeuthis dux*	2683 Mb	7376	32.9 Mb	0.16	4.85 Mb	5.7 Kb	88.50%
Cephalopoda	*Octopus bimaculoides*	2338 Mb	151,674	4.1 Mb	0.1513	475 Kb	6.6 Kb	90.30%
Brachiopoda	*Lingula anatina*	406 Mb	2677	2.17 Mb	0.0426	460 Kb	58.2 Kb	94.60%
Nemertea	*Notospermus geniculatus*	859 Mb	11,108	1.58 Mb	0.118	239 Kb	23.6 Kb	95.80%
Phoronida	*Phoronis australis*	498 Mb	3984	4.87 Mb	0.021	655 Kb	71.4 Kb	96.40%
Annelida	*Capitella teleta*	334 Mb	21,042	1.62 Mb	0.17	188 Kb	21.9 Kb	96.60%
Annelida	*Lamellibrachia luymesi*	688 Mb	11,870	2.12 Mb	0.125	373Kb	25.3Kb	95.40%
Annelida	*Helobdella robusta*	235 Mb	1993	13.6 Mb	0.0847	3.06 Mb	52.2 Kb	89.60%

Supplementary Table 3. Number of low complexity proteins in different tissues types of *Chrysomallon squamiferum*

Tissue	Scale	Mantle	Oesophageal Gland	Gill	Total
Low complexity proteins	111	212	207	342	2383
Total	723	1189	1662	2430	16917
Ratio	0.153526971	0.178301093	0.124548736	0.140741	0.140864219

Supplementary Table 4. Transcription factors involved in the hard structure formation of *Chrysomallon squamiferum* and other members of Lophotrochozoa for which data is available. Data shown for *Chrysomallon squamiferum*, conchiferan molluscs (collated from Gastropoda, Scaphopoda, Cephalopoda, Bivalvia), aculiferan molluscs (Polyplacophora data), brachiopod, and annelids. Transcription factors included are *Arx*[22,23], *Hox1/Lab*[22,24-26], *Post1*[22,24,26-28], *En*[29,30], *Hox5/Scr*[22,26,31], *Hox4/Dfd*[24,32,33], *Antp*[22], *Zic*[22], *Evx*[34], *Mox*[34], *Hox2/Pb*[26,27,32,35], *Brachyury*[23], *ETS*[23], *Dlx*[30,36], *Goosecoid*[36], *Msx*[37], *Six3/6*, *Gbx*[23,35], *Soxb2/Sox14*[38], *Grainyhead*[25], *Hox3*[26,32,35,39], *Lox4*, *Lox5*, *Pax3/7*, *Pax6*[23]. Transcription factors only verified for the Scaly-foot Snail are highlighted in red, those known to be shared across Mollusca in blue, and those shared across Lophotrochozoa in orange. Transcription factors confirmed to be absent are highlighted in grey.

Transcription Factor	Scaly-foot Snail		Conchifera					Aculifera		Brachiopoda			Annelida
	Mantle	Scale	Mantle	Proto-conch	Radula	Beak	Oper-culum	Proto-conch	Spicule	Shell	Proto-conch	Chaetae	Chaetae
Arx	+				+							+	+
Hox1/Lab	+		+	+			-	+				+	+
Post1	+		+	+								+	+
En	+			+					+		+		
Hox5/Scr	+		+								+		
Hox4/Dfd	+		+	+			+			+			
Antp		+									+		
Zic		+										+	+
Evx		+											+
Mox		+											+
Hox2/Pb		+	+										+
Brachyury	+		+	+	+								
ETS	+		+		+								
Dlx	+			+							-		
Goosecoid	+			+									
Msx	+		+										
Six3/6	+												
Gbx		+			+			+	+				
Soxb2/Sox14		+				+							
Grainyhead		+		+			+						
Hox3		+	+			+	+						
Lox4		+											
Lox5		+											
Pax3/7		+											
Pax6		+											

Supplementary Notes

Supplementary Note 1. Background information on the Scaly-foot Snail

The Kairei hydrothermal field was the first hydrothermal vent field discovered in the Indian Ocean, located and surveyed by the Japan Agency for Marine-Earth Science and Technology (JAMSTEC) R/V *KAIREI* in 2000[40]. It was later also surveyed again by the American R/V *Knorr* in 2001[41]. During these expeditions, a peculiar deep-sea gastropod mollusc living on hydrothermal vent black smoker chimneys was discovered and initially nicknamed the "scaly-foot gastropod"[42], which was later formally described as the Scaly-foot Snail *Chrysomallon squamiferum*[43]. Its foot is covered by hundreds of scales/sclerites that are mineralised with iron sulphide, mainly pyrite (FeS_2) and greigite (Fe_3S_4)[44]. Its shell is also covered by these two forms of iron sulphide, making it the only metazoan that utilizes iron in its skeleton[45]. In addition, it also has an enlarged oesophageal gland modified into a 'trophosome' harbouring a sulfur-oxidising endosymbiont[46,47], which provides energy to the host[47], and a hypertrophied circulation system to provide oxygen and hydrogen sulfide to the endosymbiont[48]. Phylogenetic analyses and anatomical characters placed *C. squamiferum* into the vent-specific gastropod family Peltospiridae[43]. Later, a second population of this species was discovered in the Solitaire field, Central Indian Ridge[49], which completely lacked iron sulfide mineralisation despite evidently being the same species as shown by both COI and nuclear SSU rRNA gene sequences[49]. This species was recently listed as one of the "Top 10 Astounding Marine Species of the Decade" selected by WoRMS (World Register of Marine Species) in April 2018, its peculiar adaptive novelties have made it one of the most charismatic deep-sea animals of all time.

Several studies to date have attempted to understand how the sclerite of the scaly-foot snail forms, and mechanisms of its biomineralisation. Initially, the scales were hypothesised to be derived from the duplication of the operculum, but the discovery of a true operculum in the scaly-foot snail and the clear differences in their underlying secretory cells rejected this explanation[50]. Instead, the great similarity between its scales and the sclerites of early Cambrian molluscs raised the possibility that the scleritome-like structures in Mollusca might undergo convergent evolution in deep time[50]. Combing physical and chemical analyses, it was revealed that crystalized pyrite and greigite are present in both inside and outside of the organic matrix of the scales[44]. Sulphur and iron isotopic analyses indicated that σ^{56} Fe values of the scales were almost the same as those

of the sulphide chimney, while σ^{34} S values of the scales were similar to those of the soft tissues of the snail. These results indicated that the iron in the scales were possibly originated from the hydrothermal fluid, and the snail may has obtained the ability to control the mineralization process by mediating the internal concentration of reduced sulphur compounds[44]. Through nanoscale experiments and computational simulations, Yao *et al.*[45] identified a multi-layered structure of the shell, and even proposed a mechanical model which may help to invent future body armour for humans. Recently, it was revealed that the animal contributes to scale biomineralisation by supplying sulfur through longitudinal, nano-scale channel-like columns in the scales which then reacts with iron ions diffusing inwards from the surrounding vent fluid[51]. Despite these morphological, physical and chemical characterisations, the underlying molecular basis of the scale formation, as well as that of the iron sulphide mineralisation, is still largely unknown.

Supplementary Note 2. Genome sequencing, assembly, and annotation

A 17-mer histogram is shown in Supplementary Figure 8. The kmer histogram was generated by with the following settings in Platanus v1.2.4 : platanus assemble -k 17 -s 10 -u 0.2 -t 29 -m 320

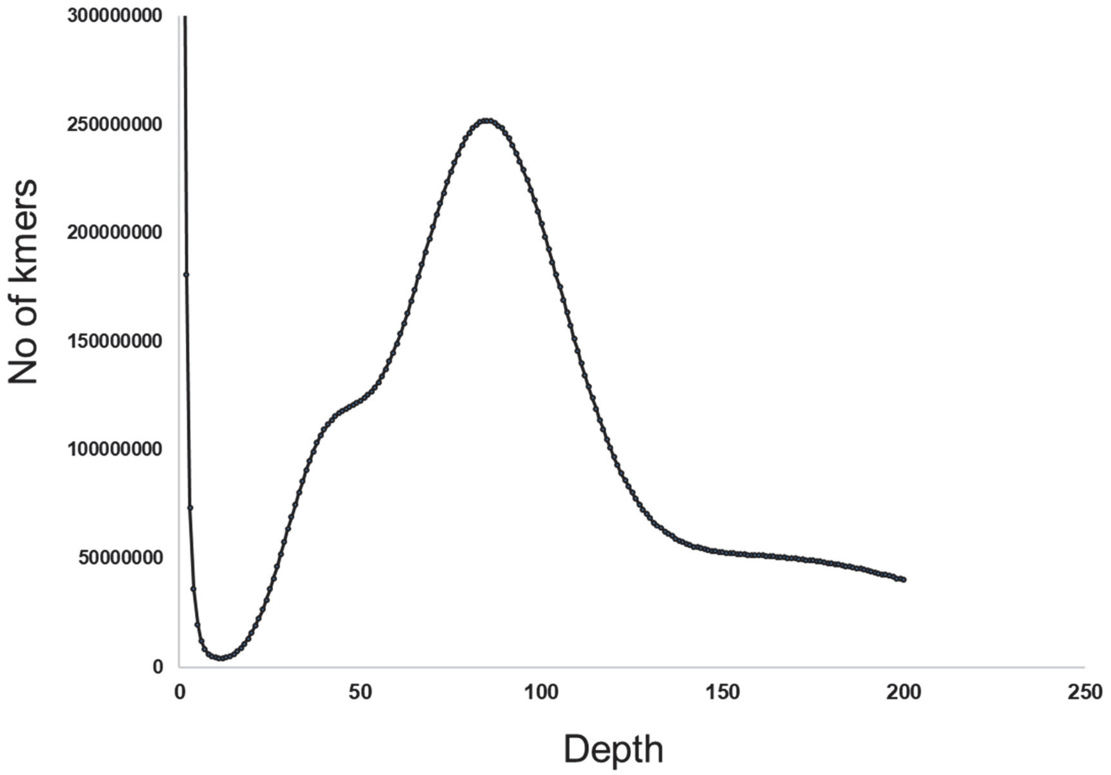

Supplementary Figure 8. A 17-mer histogram for the *Chrysomallon squamiferum* genome. Source data are provided as a Source Data file.

The detailed settings of assembly pipeline used are as follows, with assembly statistics for each summarised in Supplementary Tables 5-6.

Canu assembly

The following settings were applied to run the canu version 1.7 pipeline: "-fast genomeSize=444m corMaxEvidenceErate=0.22 corMhapSensitivity=high corMinCoverage=0 'corMhapOptions=--threshold 0.8 --num-hashes 512 --ordered-sketch-size 1000 --ordered-kmer-size 14'".

Canu + smartdenovo assembly

The corrected and trimmed ONT reads that were generated from canu version 1.7, were assembled by smartdenovo (https://github.com/ruanjue/smartdenovo), which was suggested to be very effective for ONT reads[52]. The corrected and trimmed ONT reads were approximately 40X of the genome, while the longest, N50 and average read length was 85.1Kb, 12.6Kb, and 12.6Kb, respectively. The following settings were applied to the smartdenovo assembly: -J 3000 and -c 1.

Minimap2 + miniasm assembly

The raw ONT reads were self-aligned with each other with minimap2.1, and the resultant *.paf* file was assembled with miniasm version 0.3-r179[53]. The following commands were used:

```
minimap2 -X -x ava-ont ONT_Clean.fa ONT_Clean.fa > reads.paf
miniasm -f ONT_Clean.fa reads.paf > SFS_default.gfa
awk '/^S/{print ">"$2"\n"$3}' SFS_default.gfa | fold > SFS_default.fa
```

MaSuRCA assembly

The Illumina reads and the corrected and trimmed ONT reads were also assembled by MaSuRCA pipleline (version 3.2.6). The mean and standard deviation of the Illumina library insert size was estimated from the former Platanus assembling, which was 420bp and 46bp, respectively. These were used in the MaSuRCA configuration file. The rest of the parameters retained default settings.

Supplementary Table 5. The data output from the ONT sequencing.

Flowcell No.	N50 (kb)	N25 (kb)	Longest (kb)	Output (Gb)
1	6.0	9.2	67.7	3.7
2	6.1	9.1	70.0	4.3
3	6.5	8.48	91.0	2.8
4	7.5	12.3	95.0	5.4
5	6.9	9.4	106.9	6.0
6	4.2	6.8	84.7	3.4
7	5.3	8.4	80.0	8.0
8	7.5	11.9	119.0	7.3
9	7.4	11.9	109.1	6.6
10	5.4	8.7	100.0	12.7

Supplementary Table 6. Comparison of assembly statistics by different assembling pipelines. The font in bold indicate the best score for each parameter.

Assembler	canu	smartdenovo	Canu+smartdenovo	Minimap2+miniasm	MaSuRCA
Total size (Mb)	530.8	**420.6**	399.1Mb	460.1	616.9
No of contigs	6222	1586	**1036**	3306	11,789
N50/NG50	330.0Kb/475.1Kb	1.13Mb/1.00Mb	**1.85Mb/1.68Mb**	880.7Kb/959.5Kb	468.1Kb/902.3Kb
L50/LG50	360/249	101/112	**53/66**	129/121	262/131
Longest contig	3.29Mb	5.88Mb	**10.67Mb**	8.64Mb	5.51Mb
Mean size	85.3Kb	265.2Kb	**385.2Kb**	139.2Kb	52.3Kb

Racon error correction commands:

```
minimap2 -t 80 -x map-ont SFS_canu_smartdenovo.fa ONT_clean.fq > Racon_reads.paf
racon -t 80 --bq -1 ONT_clean.fq Racon_reads.paf SFS_canu_smartdenovo.fa SFS_canu_smartdenovo_Racon.fa
```

Pilon error correction commands:

```
bowtie2-build -f SFS_canu+smartdenovo_Racon5th.fa SFS --threads 12
bowtie2 -p 80 -D 20 -R 2 -N 1 -L 18 -i S,1,0.50 --maxins 1200 -x SFS -1 SFS_500bp_trim_1.fq -2 SFS_500bp_trim_2.fq 1>SFSPE500.sam 2> SFSPE500.err
grep -E "@|NM:" SFSPE500.sam | grep -v "XS:" > SFSPE500_uniq.sam
samtools view -bS SFSPE500_uniq.sam > SFSPE500_uniq.bam -@ 8
samtools sort SFSPE500_uniq.bam -m 5G -@ 8 > SFSPE500_uniq_sorted.bam
java -jar picard.jar MarkDuplicates I= SFSPE500_uniq_sorted.bam O= SFSPE500_uniq_sorted_dedupe.bam METRICS_FILE=metrics.txt
samtools index SFSPE500_uniq_sorted_dedupe.bam
java -Xmx1200G -jar pilon-1.13.jar --genome SFS_canu+smartdenovo_Racon5th.fa --frags SFSPE500_uniq_sorted_dedupe.bam --diploid --threads 80
```

Microbial sequence contamination removal

Sequences removed from the downstream analyses are summarised as follows, in Supplementary Table 7.

Supplementary Table 7. Genome information of the two bacterial sequence sources removed.

**Genomes**	**Phylum**	**Genome size (Mb)**	**# of ORFs**	**GC content (%)**	**Completeness (%)**	**Potential contamination (%)**
Gammaproteobacterium sp.	Proteobacteria	2.62	2624	65.05	98.90	1.51
Mollicutes sp.	Tenericutes	0.77	1342	26.39	67.07	1.88

Quast genome assembly assessment report

Supplementary Table 8 shows the Quast genome assembly assessment report.

Supplementary Table 8. Quast genome assembly assessment report.

**Assembly**	**Csq_v1.1**
No. of contigs (>= 50000 bp)	15
Total length (>= 25000 bp)	404517194
Total length (>= 50000 bp)	404477230
No of contigs	22
Largest contig	49219174
Total length	404615235
GC (%)	29.83
N50	30197626
N75	19579237
L50	6
L75	10
ONT reads mapped (%)	97.79
Avg. coverage depth	139
Coverage >= 1X (%)	99.95
Coverage >= 5X (%)	99.93
Coverage >= 10X (%)	99.91
N's per 100 kbp	51.22

Repeats annotation

Composition of repeats as summarized and classified with the perl script buildSummary.pl in the RepeatMasker package is presented in Supplementary Table 9.

Supplementary Table 9. Composition of repeats in the *Chrysomallon squamiferum* genome.

Repeats	Total length	Percentage
**DNA transposons**	3007700	0.74%
CMC-EnSpm	14920821	3.69%
Crypton	218536	0.05%
Dada	498341	0.12%
Ginger	707657	0.17%
Kolobok-T2	82506	0.02%
Maverick	31629	0.01%
MuLE-MuDR	272936	0.07%
Novosib	213863	0.05%
Sola	1318507	0.33%
TcMar	54915	0.01%
TcMar-Pogo	67399	0.02%
TcMar-Tc1	471827	0.12%
TcMar-Tigger	2965145	0.73%
hAT	116766	0.03%
hAT-Ac	302426	0.07%
hAT-Charlie	405093	0.10%
hAT-Tip100	264422	0.07%
hAT-hAT5	44955	0.01%
hAT-hATw	41774	0.01%
**LINE**		
I	259919	0.06%
I-Nimb	565481	0.14%
Jockey	163075	0.04%
L1	303512	0.08%
L1-Tx1	963469	0.24%
L2	1673121	0.41%
Penelope	776740	0.19%
R1	81075	0.02%

RTE-BovB	16045	0.00%
RTE-X	717790	0.18%
Tad1	52348	0.01%
LTR		
Copia	263030	0.07%
ERV1	108196	0.03%
Gypsy	1752986	0.43%
Gypsy-Cigr	203276	0.05%
Ngaro	211466	0.05%
Pao	449984	0.11%
**RC** - Helitron	118857	0.03%
**SINE**	9339	0.00%
Alu	52908	0.01%
ID	8456	0.00%
**Unknown**	18401439	4.55%
**Low_complexity**	6117114	1.51%
**Satellite**	68819	0.02%
**Simple_repeat**	42666879	10.55%
**snRNA**	20553	0.01%
**Total**	102033095	25.22%

Supplementary Note 3. Chromosomal distribution of genes

Chromosomal distribution of genes in the Scaly-foot Snail genome was examined using a hypergeometric test (Supplementary Table 10).

Supplementary Table 10. The chromosome distribution of the genes in the expanded gene families.

Chr	No of expanded genes in each chr	No of genes in each chr	Ratio	Enriched fold-change	FDR	Over/under represented
chr1	155	2113	0.07336	1.91	1.4E-23	-
chr2	71	658	0.1079	1.3	0.00937	-
chr3	162	1049	0.15443	1.1	0.09786	+
chr4	141	583	0.24185	1.73	3.2E-11	+
chr5	91	806	0.1129	1.24	0.01328	-
chr6	160	1901	0.08417	1.66	6E-15	-
chr7	158	1773	0.08911	1.57	8.5E-12	-
chr8	176	1559	0.11289	1.24	0.0007	-
chr9	102	1369	0.07451	1.88	8.1E-15	-
chr10	103	700	0.14714	1.05	0.30657	+
chr11	262	776	0.33763	2.41	1E-45	+
chr12	259	665	0.38947	2.78	1.1E-58	+
chr13	203	737	0.27544	1.03	2E-22	+
chr14	178	822	0.21655	1.55	1.1E-09	+
chr15	147	1403	0.10478	1.34	3.6E-05	-
Total	2369	16917	0.14003			

Supplementary Note 4. Transcriptome Gene Ontology enrichment analyses

Gene Ontology (GO) enrichment analyses using GOEAST[54] or in the BLAST2GO package, with results shown as follows (Supplementary Figures 9-12).

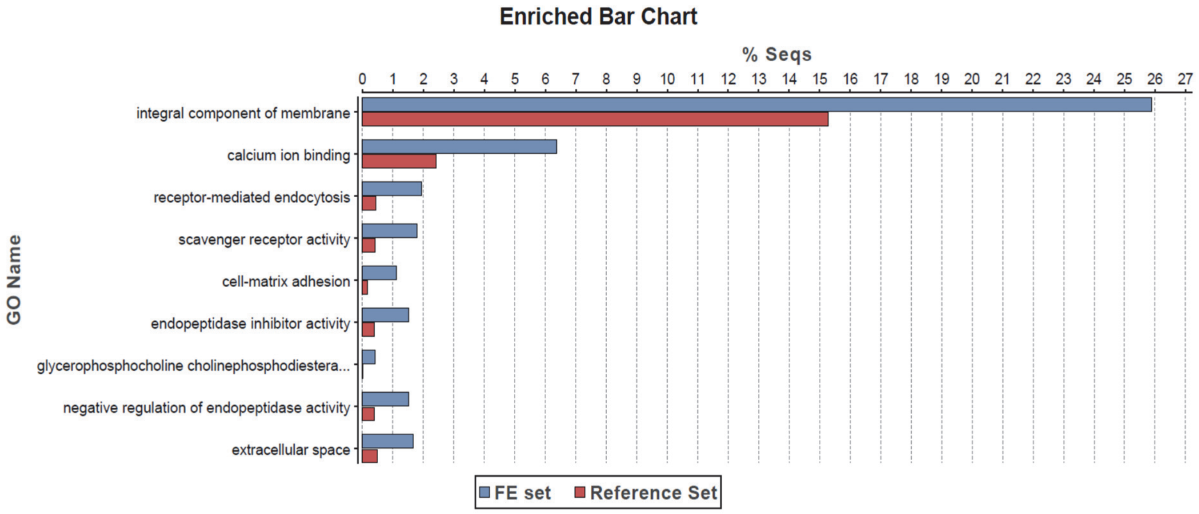

Supplementary Figure 9. GO enrichment analysis on highly expressed genes in the scales. Source data are provided as a Source Data file.

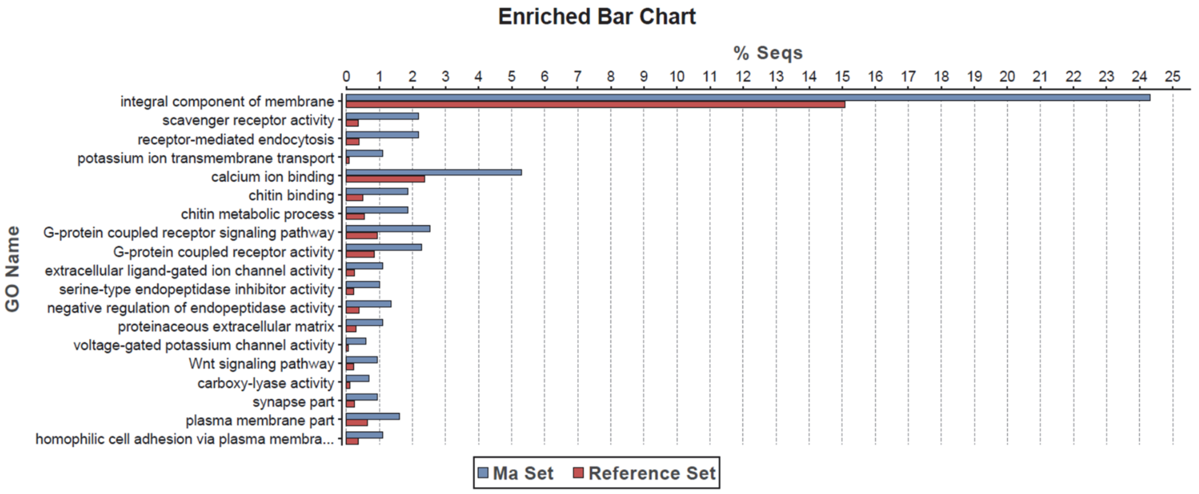

Supplementary Figure 10. GO enrichment analysis on highly expressed genes in the mantle. Source data are provided as a Source Data file.

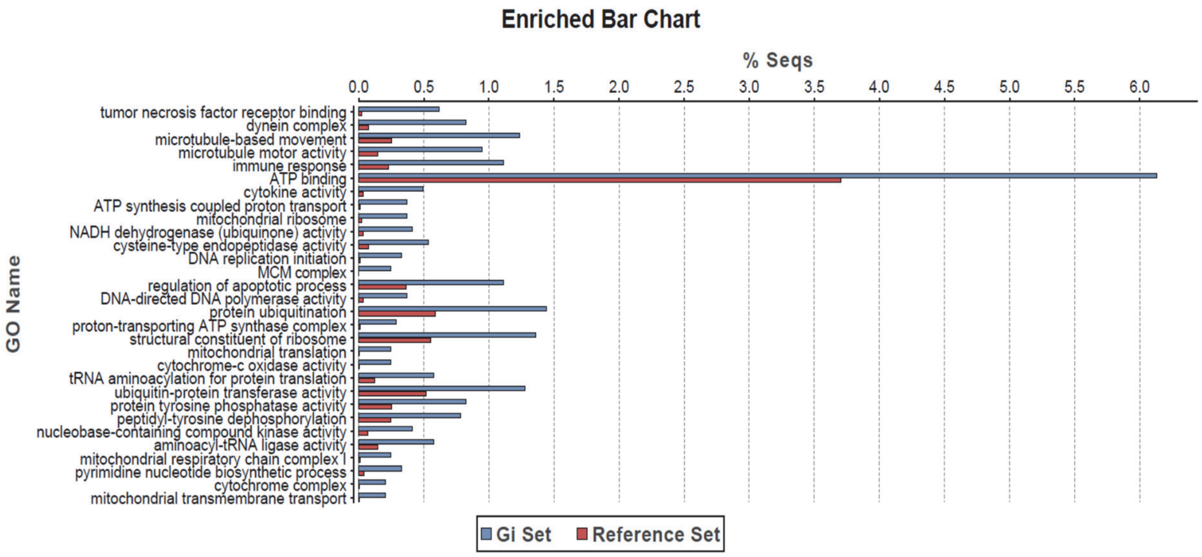

Supplementary Figure 11. GO enrichment analysis on highly expressed genes in the oesophageal gland. Source data are provided as a Source Data file.

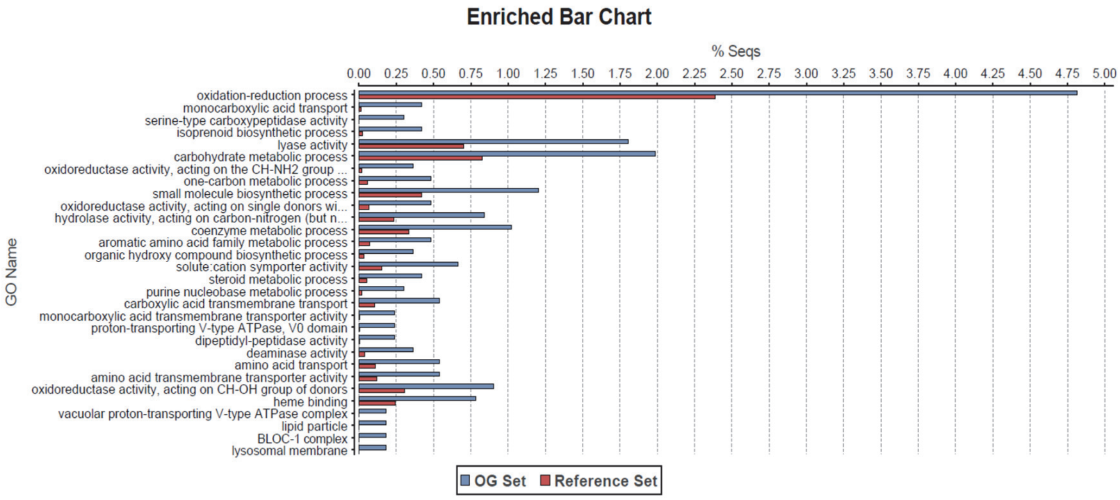

Supplementary Figure 12. GO enrichment analysis on highly expressed genes in the gill. Source data are provided as a Source Data file.

Supplementary Note 5. Real-time PCR validation

The primers for real-time PCR (Supplementary Table 11) were designed using the online NCBI Primer-BLAST tool (https://www.ncbi.nlm.nih.gov/tools/primer-blast/).

Supplementary Table 11. Primers that were used for the real-time PCR analysis.

Gene ID	Annotation	Forward primer	Reverse primer
CsqKR_Scaf285_2.27	Pax 3/7	CAGGAGGTGAGGCAGACAAG	GCTCCGCTGTGAAGGTAGTT
CsqKR_Scaf153_1.11	GBX	CCAACTCGGACTCTGTCCAC	ATTGTGCGCGATATGAGAGC
CsqKR_Scaf379_3.12	Hox3	ACGGAAGGGAATTGTGGGTC	AATGTGACGACTGCTGAGGG
CsqKR_Scaf89_2.6	Lox5	GAGGCCTCGGGAAGTTGAAA	GCCTTCTGGTCAGGTATCGG
CsqKR_Scaf41_38.19	Grainyhead	GTACGACCAGTTCACCGAGG	CTCGGGTCGAGTTGGAACAT
CsqKR_Scaf379_3.14	Hox2	GTTACGGGACACTACGGCAA	CCGTTGCACTGAACCTCTCT
CsqKR_Scaf84_7.8	Chitin synthase	TCTGACGCGACTTTGGTTGA	CTGGTACTGCACCACTCCTG
CsqKR_Scaf191_15.3	ZIC 4	CGAACCGAGGAAACCGTGTA	GCGCAGTTTTGCCAGTAACA
CsqKR_Scaf99_59.6	Pax-6	GCCAGACTCGACGAGACAAA	GGATCTTGGAGACACACCCG
CsqKR_Scaf89_4.10	Lox4	GTAGACGAGGTCGCCAAACT	CTGTCTCTCCGTCAAGCACA
CsqKR_Scaf112_6.3	Mox-2	CAAACTCGACCTGTCGGTGA	GCTCGTAACGACGTAAACGC
CsqKR_Scaf201_1.11	EVX	GGAGCAGATCGGAAGACTGG	TCTTTGATCGTGCTCTCGGG
CsqKR_Scaf89_3.8	Antp	GTCTTGCGCAACAGGATTGG	AGAACAGCGTTCGGCCATAA
CsqKR_Scaf269_6.5	SoxB2	ATGACTTCTCAGCCCATGCC	CGTGGTCGTTGTTGCTCTTG
CsqKR_Scaf130_17.25	Metal tolerance protein	TCGACAGAGTCAGTGCAACC	CTGAGTGACGGCGATGGAAT
CsqKR_Scaf182_4.8	EF1a	GATTCCATCCTGCCACCCAA	AACTCCGGTCTCAACACGAC

Results of real-time PCR validations are shown in Supplementary Figure 13.

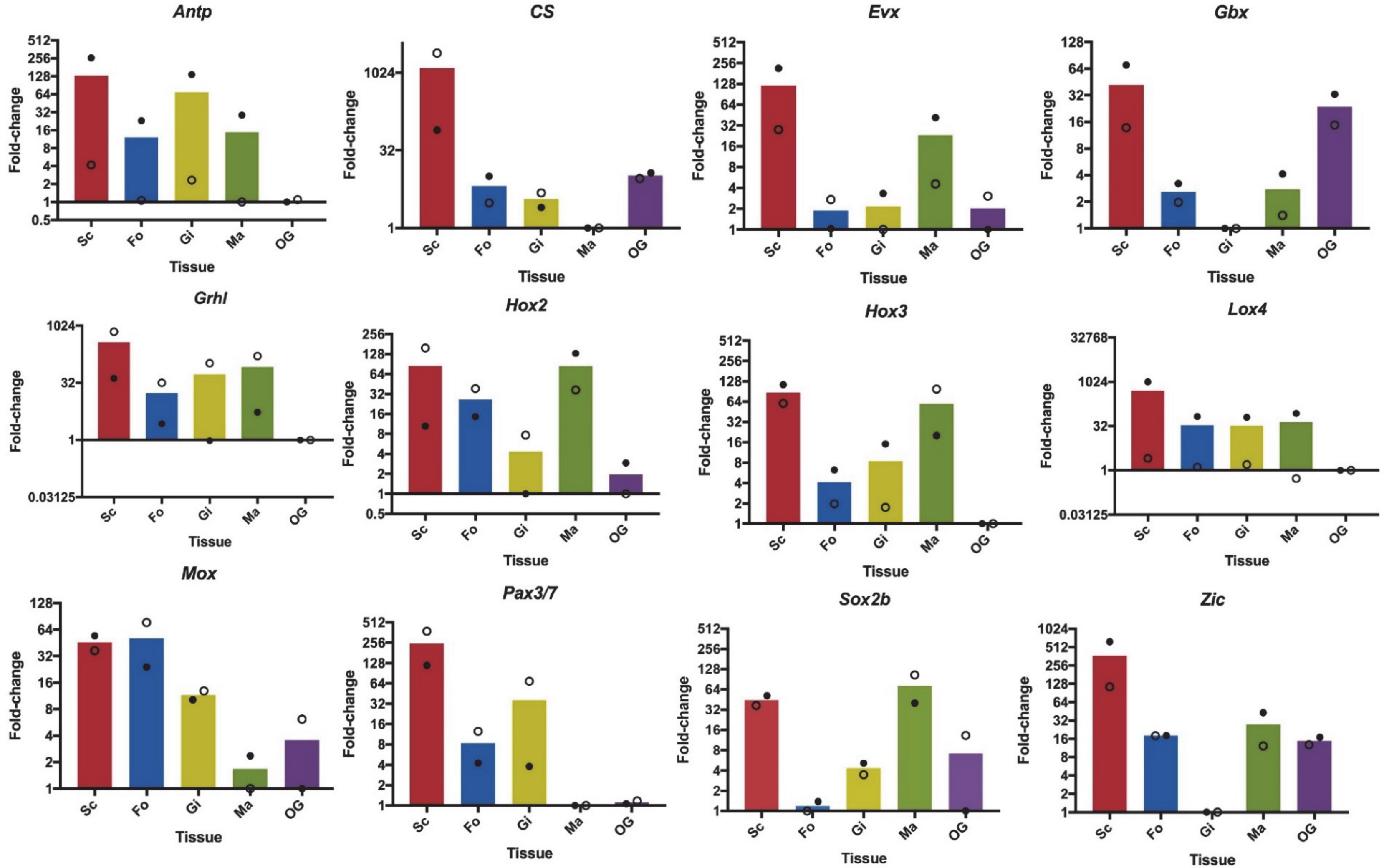

Supplementary Figure 13. Real-time PCR results showing gene expression patterns among five tissues (*n* = 2 biologically independent animals): FE, scales; ME, mantle; FI, foot muscle; OG, oesophageal gland; Gi, gill. The y-axis was $\log_2$ scaled. Dots displayed in the picture indicate the fold-change of particular gene expression, open dots and closed dots indicate values of biological sample acquired from Kairei (B2) and Solitaire (IW2) fields, respectively. For the gene *Lox5*, RNA expression level was only detected as highly expressed in a single tissue type, the scales, and therefore was not included. CS, chitin synthase. Source data are provided as a Source Data file.

Supplementary Note 6. Gene family and phylogenetic analyses

The orthologue groups (OGs) were determined by a BLASTp search against protein sequences of other molluscan, lophotrochozoan, and metazoan genomes. The full list of species used (in alphabetical order) and their sources are as follows:

Acropora digitifera[55]
Aplysia californica (https://www.ncbi.nlm.nih.gov/assembly/GCF_000002075.1/)
Aurelia sp.[56]
Azumapecten farreri[11]
Bathymodiolus platifrons[14]
Biomphalaria glabrata[5]
Branchiostoma floridae[57]
Capitella teleta[6]
Crassostrea gigas[10]
Danio rerio (ftp://ftp.ensembl.org/pub/release-89/fasta/danio_rerio/pep/)
Daphnia pulex[58]
Drosophila melanogaster (ftp://ftp.ensembl.org/pub/release-89/fasta/drosophila_melanogaster/pep/)
Haliotis discus hannai[8]
Homo sapiens (ftp://ftp.ensembl.org/pub/release-89/fasta/homo_sapiens/pep/)
Lanistes nyassanus[59]
Lingula anatina[33]
Lottia gigantea[6]
Modiolus philippinarum[14]
Nematostella vectensis (https://genome.jgi.doe.gov/Nemve1/Nemve1.home.html)
Notospermus geniculatus[20]
Octopus bimaculoides[17]
Mizuhopecten yessoensis[2]
Pinctada fucata[15]
Phoronis australis[20]
Pomacea canaliculata[59]
Radix auricularia[4]

Supplementary References

1 Hui, J. H. L. *et al.* Extensive chordate and annelid macrosynteny reveals ancestral homeobox gene organization. *Mol. Biol. Evol.* 29, 157-165 (2011).

2 Wang, S. *et al.* Scallop genome provides insights into evolution of bilaterian karyotype and development. *Nature Ecol. Evol.* 1, 0120 (2017).

3 Liu, C. *et al.* The genome of the golden apple snail *Pomacea canaliculata* provides insight into stress tolerance and invasive adaptation. *GigaScience* 7 (2018).

4 Schell, T. *et al.* An annotated draft genome for *Radix auricularia* (Gastropoda, Mollusca). *Genome Biol. Evol.* 9, 0-0 (2017).

5 Adema, C. M. *et al.* Whole genome analysis of a schistosomiasis-transmitting freshwater snail. *Nature Communications* 8, 15451 (2017).

6 Simakov, O. *et al.* Insights into bilaterian evolution from three spiralian genomes. *Nature* 493, 526 (2012).

7 Cai, H. *et al.* A draft genome assembly of the solar-powered sea slug *Elysia chlorotica*. *Scientific Data* 6, 190022 (2019).

8 Nam, B.-H. *et al.* Genome sequence of pacific abalone (*Haliotis discus hannai*): the first draft genome in family Haliotidae. *GigaScience* 6 (2017).

9 Guo, Y. *et al.* A chromosomal-level genome assembly for the giant African snail *Achatina fulica*. *GigaScience* 8 (2019).

10 Zhang, G. *et al.* The oyster genome reveals stress adaptation and complexity of shell formation. *Nature* 490, 49 (2012).

11 Li, Y. *et al.* Scallop genome reveals molecular adaptations to semi-sessile life and neurotoxins. *Nature Communications* 8, 1721 (2017).

12 Ran, Z. *et al.* Chromosome-level genome assembly of the razor clam *Sinonovacula constricta* (Lamarck, 1818). *Mol. Ecol. Resour.* 19, 1647-1658 (2019).

13 Yan, X. *et al.* Clam genome sequence clarifies the molecular basis of its benthic adaptation and extraordinary shell color diversity. *iScience* 19, 1225-1237 (2019).

14 Sun, J. *et al.* Adaptation to deep-sea chemosynthetic environments as revealed by mussel genomes. *Nature Ecol. Evol.* 1, 0121 (2017).

15 Takeuchi, T. *et al.* Bivalve-specific gene expansion in the pearl oyster genome: implications of adaptation to a sessile lifestyle. *Zool. Lett.* 2, 3 (2016).

16 da Fonseca, R. R. *et al.* A draft genome sequence of the elusive giant squid, *Architeuthis dux*. *GigaScience* 9 (2020).

17 Albertin, C. B. *et al.* The octopus genome and the evolution of cephalopod neural and morphological novelties. *Nature* 524, 220 (2015).

18 Belcaid, M. *et al.* Symbiotic organs shaped by distinct modes of genome evolution in cephalopods. *Proceedings of the National Academy of Sciences* 116, 3030-3035 (2019).

19 Gerdol, M., Luo, Y.-J., Satoh, N. & Pallavicini, A. Genetic and molecular basis of the immune system in the brachiopod *Lingula anatina*. *Dev Comp Immunol* 82, 7-30 (2018).

20 Luo, Y.-J. *et al.* Nemertean and phoronid genomes reveal lophotrochozoan evolution and the origin of bilaterian heads. *Nature Ecology & Evolution* 2, 141-151 (2018).

21 Li, Y. *et al.* Genomic adaptations to chemosymbiosis in the deep-sea seep-dwelling tubeworm *Lamellibrachia luymesi*. *BMC Biol* 17, 91 (2019).

22 Schiemann, S. M. *et al.* Clustered brachiopod Hox genes are not expressed collinearly and are associated with lophotrochozoan novelties. *Proc. Natl. Acad. Sci.* 114, E1913-E1922 (2017).

23 Hilgers, L., Hofreiter, M., Hartmann, S. & von Rintelen, T. Novel genes, ancient genes, and gene co-option contributed to the genetic basis of the radula, a molluscan innovation. *Mol. Biol. Evol.* 35, 1638-1652 (2018).

24 Samadi, L. & Steiner, G. Involvement of Hox genes in shell morphogenesis in the encapsulated development of a top shell gastropod (*Gibbula varia* L.). *Dev Genes Evol* 219, 523-530 (2009).

25 Hashimoto, N., Kurita, Y. & Wada, H. Developmental role of dpp in the gastropod shell plate and co-option of the dpp signaling pathway in the evolution of the operculum. *Dev Biol* 366, 367-373 (2012).

26 Wollesen, T., Rodriguez Monje, S. V., Luiz de Oliveira, A. & Wanninger, A. Staggered Hox expression is more widespread among molluscs than previously appreciated. *Proc. Biol. Sci.* 285 (2018).

27 Kulakova, M. *et al.* Hox gene expression in larval development of the polychaetes *Nereis virens* and *Platynereis dumerilii* (Annelida, Lophotrochozoa). *Dev Genes Evol* 217, 39-54 (2007).

28 Fröbius, A. C., Matus, D. Q. & Seaver, E. C. Genomic organization and expression demonstrate spatial and temporal Hox gene colinearity in the lophotrochozoan *Capitella* sp. I. *PLoS One* 3, e4004-e4004 (2008).

29 Jacobs, D. K. *et al.* Molluscan engrailed expression, serial organization, and shell evolution. *Evol Dev* 2, 340-347 (2000).

30 Shimizu, K., Luo, Y.-J., Satoh, N. & Endo, K. Possible co-option of *engrailed* during brachiopod and mollusc shell development. *Biol Lett* 13, 20170254 (2017).

31 Gąsiorowski, L. & Hejnol, A. Hox gene expression in postmetamorphic juveniles of the brachiopod *Terebratalia transversa*. *EvoDevo* 10, 1-1 (2019).

32 Hinman, V. F., O'Brien, E. K., Richards, G. S. & Degnan, B. M. Expression of anterior Hox genes during larval development of the gastropod *Haliotis asinina*. *Evol Dev* 5, 508-521 (2003).

33 Luo, Y.-J. *et al.* The *Lingula* genome provides insights into brachiopod evolution and the origin of phosphate biomineralization. *Nature Communications* 6, 8301 (2015).

34 Kozin, V. V., Filimonova, D. A., Kupriashova, E. E. & Kostyuchenko, R. P. Mesoderm patterning and morphogenesis in the polychaete *Alitta virens* (Spiralia, Annelida): Expression of mesodermal markers Twist, Mox, Evx and functional role for MAP kinase signaling. *Mech Dev* 140, 1-11 (2016).

35 Wollesen, T. *et al.* Brain regionalization genes are co-opted into shell field patterning in Mollusca. *Sci. Rep.* 7, 5486 (2017).

36 Jackson, D. J. & Degnan, B. M. The importance of evo-devo to an integrated understanding of molluscan biomineralisation. *J Struct Biol* 196, 67-74 (2016).

37 Zhao, M., He, M., Huang, X. & Wang, Q. A homeodomain transcription factor gene, PfMSX, activates expression of Pif gene in the pearl oyster *Pinctada fucata*. *PLoS One* 9, e103830-e103830 (2014).

38 Focareta, L. & Cole, A. G. Analyses of Sox-B and Sox-E family genes in the cephalopod *Sepia officinalis*: revealing the conserved and the unusual. *PLoS One* 11, e0157821 (2016).

39 Focareta, L., Sesso, S. & Cole, A. G. Characterization of Homeobox genes reveals sophisticated regionalization of the central nervous system in the European Cuttlefish *Sepia officinalis*. *PLoS One* 9, e109627 (2014).

40 Hashimoto, J. *et al. First hydrothermal vent communities from the Indian Ocean discovered*. Vol. 18 (Zoological Science, 2001).

41 Van Dover, C. L. *et al.* Biogeography and ecological setting of Indian Ocean hydrothermal vents. *Science* 294, 818-823 (2001).

42 Warén, A., Bengtson, S., Goffredi, S. K. & Van Dover, C. L. A hot-vent gastropod with iron sulfide dermal sclerites. *Science* 302, 1007-1007 (2003).

43 Chen, C., Linse, K., Copley, J. T. & Rogers, A. D. The ‘scaly-foot gastropod’: a new genus and species of hydrothermal vent-endemic gastropod (Neomphalina: Peltospiridae) from the Indian Ocean. *J Molluscan Stud* 81, 322-334 (2015).

44 Suzuki, Y. *et al.* Sclerite formation in the hydrothermal-vent “scaly-foot” gastropod—possible control of iron sulfide biomineralization by the animal. *Earth. Planet. Sci. Lett.* 242, 39-50 (2006).

45 Yao, H. *et al.* Protection mechanisms of the iron-plated armor of a deep-sea hydrothermal vent gastropod. *Proc. Natl. Acad. Sci.* 107, 987-992 (2010).

46 Goffredi, S. K., Waren, A., Orphan, V. J., Van Dover, C. L. & Vrijenhoek, R. C. Novel forms of structural integration between microbes and a hydrothermal vent gastropod from the Indian Ocean. *Appl Environ Microbiol* 70, 3082-3090 (2004).

47 Nakagawa, S. *et al.* Allying with armored snails: the complete genome of gammaproteobacterial endosymbiont. *The ISME journal* 8, 40-51 (2014).

48 Chen, C., Copley, J. T., Linse, K., Rogers, A. D. & Sigwart, J. D. The heart of a dragon: 3D

anatomical reconstruction of the 'scaly-foot gastropod' (Mollusca: Gastropoda: Neomphalina) reveals its extraordinary circulatory system. *Front Zool* 12, 13 (2015).

49 Nakamura, K. *et al.* Discovery of new hydrothermal activity and chemosynthetic fauna on the Central Indian Ridge at 18°–20°S. *PLoS One* 7, e32965 (2012).

50 Chen, C., Copley, J. T., Linse, K., Rogers, A. D. & Sigwart, J. How the mollusc got its scales: convergent evolution of the molluscan scleritome. *Biol J Linn Soc* 114, 949-954 (2015).

51 Okada, S. *et al.* The making of natural iron sulfide nanoparticles in a hot vent snail. *Proc. Natl. Acad. Sci. U. S. A.*, DOI: 10.1073/pnas.1908533116 (2019).

52 Schmidt, M. H.-W. *et al.* De Novo assembly of a new *Solanum pennellii* accession using nanopore sequencing. *The Plant Cell* 29, 2336-2348 (2017).

53 Li, H. Minimap and miniasm: fast mapping and de novo assembly for noisy long sequences. *Bioinformatics* 32, 2103-2110 (2016).

54 Zheng, Q. & Wang, X. J. GOEAST: a web-based software toolkit for Gene Ontology enrichment analysis. *Nucleic Acids Res* 36, W358-363 (2008).

55 Shinzato, C. *et al.* Using the *Acropora digitifera* genome to understand coral responses to environmental change. *Nature* 476, 320 (2011).

56 Gold, D. A. *et al.* The genome of the jellyfish *Aurelia* and the evolution of animal complexity. *Nature Ecology & Evolution* 3, 96-104 (2019).

57 Putnam, N. H. *et al.* The amphioxus genome and the evolution of the chordate karyotype. *Nature* 453, 1064-1071 (2008).

58 Colbourne, J. K. *et al.* The ecoresponsive genome of *Daphnia pulex*. *Science* 331, 555-561 (2011).

59 Sun, J. *et al.* Signatures of divergence, invasiveness and terrestralization revealed by four apple snail genomes *Mol. Biol. Evol.* 36, 1507-1520 (2019).
