# Supplementary material for: The Scaly-foot Snail genome and implications for the origins of biomineralised armour
Source: Nat Commun. 2020 Apr 8;11:1657. doi: 10.1038/s41467-020-15522-3 (PMC7142155; doi:10.1038/s41467-020-15522-3)
Supplement: Supplementary file 3 — Description of Additional Supplementary Information [file 41467_2020_15522_MOESM3_ESM.pdf]

Description of Additional Supplementary Files

File Name: Supplementary Data 1
Description: Expanded gene families in *Chrysomallon squamiferum*, compared to other lophotrochozoan genomes.

File Name: Supplementary Data 2
Description: pfam domain distribution on each DMBT1 gene copies in *Chrysomallon squamiferum*.

File Name: Supplementary Data 3
Description: Annotation of low complexity proteins in *Chrysomallon squamiferum*

File Name: Supplementary Data 4
Description: Contracted gene families in *Chrysomallon squamiferum*, compared to other lophotrochozoan genomes.

File Name: Supplementary Data 5
Description: Genes highly expressed in the scale-secreting epithelium of *Chrysomallon squamiferum.*

File Name: Supplementary Data 6
Description: Genes highly expressed in the mantle of *Chrysomallon squamiferum*.

File Name: Supplementary Data 7
Description: Genes highly expressed in the oesophageal gland of *Chrysomallon squamiferum*.

File Name: Supplementary Data 8
Description: Comparison of gene expression levels between individuals of *Chrysomallon squamiferum* from Kairei and Solitaire vent fields.

File Name: Supplementary Data 9
Description: Text file including the following components: 1) All commands used in this study; 2) OrthoMCL results; 3) The summary and classification of the repeats; and 4) The input sequences (phylip format) for the MCMCTree analysis.
