# Supplementary material for: The Scaly-foot Snail genome and implications for the origins of biomineralised armour
Source: Nat Commun. 2020 Apr 8;11:1657. doi: 10.1038/s41467-020-15522-3 (PMC7142155; doi:10.1038/s41467-020-15522-3)
Supplement: Supplementary file 13 — Reporting summary [file 41467_2020_15522_MOESM13_ESM.pdf]

nature research

Corresponding author(s): Ken Takai / Pei-Yuan Qian

Last updated by author(s): Mar 5, 2020

Reporting Summary

Nature Research wishes to improve the reproducibility of the work that we publish. This form provides structure for consistency and transparency in reporting. For further information on Nature Research policies, see Authors & Referees and the Editorial Policy Checklist .

Statistics

For all statistical analyses, confirm that the following items are present in the figure legend, table legend, main text, or Methods section.

n/a	Confirmed	
☐	☒	The exact sample size (*n*) for each experimental group/condition, given as a discrete number and unit of measurement
☐	☒	A statement on whether measurements were taken from distinct samples or whether the same sample was measured repeatedly
☐	☒	The statistical test(s) used AND whether they are one- or two-sided<br>*Only common tests should be described solely by name; describe more complex techniques in the Methods section.*
☒	☐	A description of all covariates tested
☐	☒	A description of any assumptions or corrections, such as tests of normality and adjustment for multiple comparisons
☐	☒	A full description of the statistical parameters including central tendency (e.g. means) or other basic estimates (e.g. regression coefficient) AND variation (e.g. standard deviation) or associated estimates of uncertainty (e.g. confidence intervals)
☐	☒	For null hypothesis testing, the test statistic (e.g. *F*, *t*, *r*) with confidence intervals, effect sizes, degrees of freedom and *P* value noted<br>*Give P values as exact values whenever suitable.*
☒	☐	For Bayesian analysis, information on the choice of priors and Markov chain Monte Carlo settings
☒	☐	For hierarchical and complex designs, identification of the appropriate level for tests and full reporting of outcomes
☒	☐	Estimates of effect sizes (e.g. Cohen's *d*, Pearson's *r*), indicating how they were calculated

Our web collection on statistics for biologists contains articles on many of the points above.

Software and code

Policy information about availability of computer code

Data collection	MinKNOW 1.3.23 was used for the Oxford Nanopore Technologies read collection. Illumina Novaseq platform with the paired-end mode and a read length of 150bp was used for Hi-C sequencing and transcriptome sequencing.
Data analysis	Software used in data analyses in the present study are listed as follows:<br><br>Albacore v2.3.4, Trimmomatic v0.33, Platanus v1.2.4, GenomeScope (http://qb.cshl.edu/genomescope/), canu version 1.7, smartdenovo (https://github.com/ruanjue/smartdenovo), MaSuRCA version 3.2.6, minimap2 version 2.17-r943-dirty, miniasm version 0.3-r179, Racon v1.4, Pilon v1.13, BUSCO v3.0, MetaBAT v2, CheckM v1.0.7, Prodigal v2.6.3, MEGA 6, MEGAN 6, HiC-Pro v2.10.0, Juicer 1.5.6, Quast v4.0, 3D de novo assembly pipeline (https://github.com/theaidenlab/3d-dna), RepeatModeler 2.0, RepeatMasker 4.0.8, RMBlast v2.9.0, Trinity v2.6.5, histat2 v2.1.0, PASA v2.2.0, cd-hit-est v4.6, Maker v3.0, RAxML v8.2.4, FigTree v1.4.3, ProtTest v3.2, CAFÉ v3.1, Kallisto v0.44.0, GOEAST v1.30, and BLAST2GO pro v4.1.9, KEGG Automatic Annotation Server (https://www.genome.jp/kegg/kaas/), XSTREAM v1.73, OrthoMCL v2.0.9, MCMCtree in the Phylogenetic Analysis by Maximum Likelihood (PAML) package v4.9 (http://abacus.gene.ucl.ac.uk/software/paml.html), R 3.5.2.<br><br>Furthermore, commands and intermediate files of all analyses are included as Supplementary Data 9.

For manuscripts utilizing custom algorithms or software that are central to the research but not yet described in published literature, software must be made available to editors/reviewers. We strongly encourage code deposition in a community repository (e.g. GitHub). See the Nature Research guidelines for submitting code & software for further information.

Data

Policy information about availability of data

All manuscripts must include a data availability statement. This statement should provide the following information, where applicable:

- Accession codes, unique identifiers, or web links for publicly available datasets
- A list of figures that have associated raw data
- A description of any restrictions on data availability

The Chrysomallon squamiferum genome that support the findings of this study have been deposited in the NCBI Sequence Read Archive under the BioProject number PRJNA523462 [https://www.ncbi.nlm.nih.gov/bioproject/PRJNA523462], all raw sequencing data, including Illumina and Nanopore reads, are also deposited under the same BioProject number. The assembled genome, transcriptome, predicted transcripts, proteins have been deposited in Dryad [https://doi.org/10.5061/dryad.24053dn].

Publicly available datasets used in the study include the following: NCBI NR database (https://www.ncbi.nlm.nih.gov/refseq/), Repbase (https://www.girinst.org/repbase/), SwissProt database via UniProt (https://www.uniprot.org/), and KEGG (https://www.genome.jp/kegg/).

Field-specific reporting

Please select the one below that is the best fit for your research. If you are not sure, read the appropriate sections before making your selection.

☐ Life sciences ☐ Behavioural & social sciences ☒ Ecological, evolutionary & environmental sciences

For a reference copy of the document with all sections, see nature.com/documents/nr-reporting-summary-flat.pdf

Ecological, evolutionary & environmental sciences study design

All studies must disclose on these points even when the disclosure is negative.

Study description	This study aimed to sequence the whole genome and tissue-specific transcriptome of the enigmatic Scaly-foot Snail (Chrysomallon squamiferum), only known from deep-sea hydrothermal vents, in order to shed light on the evolutionary and genomic basis of its unique scleritome. For genome sequencing, one individual was used for the contigs assembly, and another individual was sequenced by Hi-C technique to further scaffold the contigs into pseudo-chromosomes. For the gene expression analysis, a total of five individuals (two from Kairei field and three from Solitaire field) were used.
Research sample	Taxa: Chrysomallon squamiferum (Mollusca: Gastropoda: Peltospiridae). This species is hermaphroditic. Individuals between 30 mm to 40 mm in shell length were used in the present study (age estimates not available for this deep-sea species), representing typical adult, sexually matured, individuals. Specimens used were collected from two different hydrothermal vent fields in Indian Ocean, including Kairei field (25°19.23’S, 70°02.42’E, 2415 m) and Solitaire field (19°33.41’S, 65°50.89’E, 2606 m depth).
Sampling strategy	Individuals of Chrysomallon squamiferum were collected using a slurp gun (suction sampler) mounted on the manned submersible Shinkai 6500 from two hydrothermal vent fields in the Indian Ocean, including Kairei field (25°19.23’S, 70°02.42’E, 2415 m depth) and Solitaire field (19°33.41’S, 65°50.89’E, 2606 m depth). Individuals used for gene expression analyses were fixed in situ using RNAlater solution, in order to make sure the gene expression patterns analysed reflect those in the natural condition and not influenced by the changes in pressure, temperature, and other environmental factors during the submersible's ascend and recovery to the research vessel. Individuals used for genome/Hi-C sequencing were immediately placed in -80°C freezer when the submersible was recovered to the research vessel, in order to best preserve their intact genomic DNA.<br><br>A single individual collected from Kairei field was used for genome sequencing, and another one individual was used for Hi-C sequencing. The usage of a single individual for each avoids heterozygosity issues from mixing individuals, as these are wild animals not from an inbred line. Individuals from Kairei field have scales highly mineralised with iron sulphide, while those from Solitaire lack iron sulphide. Both populations were used for transcriptome sequencing in order to investigate if associated differences are present on the transcriptomes. Three individuals from Kairei field and five individuals from Solitaire field were used, in order to have more than one individual from each population for replication purposes. The sample size used was ultimately also limited by sample availability of these rare snails.<br><br>Sampling in the Mauritian EEZ was approved by Ministry of Foreign Affairs, Regional Integration, and International Trade, Mauritian Government (Ref. 29/2014; 50/38/24 V2).
Data collection	Specimens of the Scaly-foot Snail (Chrysomallon squamiferum) used in the present study were collected by the manned submersible Shinkai 6500 on-board multiple deep-sea research expeditions of R/V Yokosuka (specimen collecting details in "Sampling strategy" section above). Data recorded by Chong Chen and Ken Takai.
Timing and spatial scale	R/V Yokosuka research cruises were carried out in the following months: cruise YK13-02, Feb 2013; cruise YK13-03, Mar 2013; cruise YK16-02E, Feb 2016. These timings are determined by 1. Funding and ship time assigned, and 2. Likelihood of fine sea conditions. Samples are taken from the following sites: Kairei field, 25°19.23’S, 70°02.42’E, 2415 m depth; Solitaire field: 19°33.41’S, 65°50.89’E, 2606 m depth.
Data exclusions	No data were excluded.

Reproducibility	For the gene expression analysis, five replicates were applied and all attempts successful. The gene expression profile was validated by real-time PCR (two individuals) and in situ hybridization (three individuals). We can confirm that all these attempts were successful.
Randomization	The samples were collected randomly in the field.
Blinding	Blinding was not possible in this study because only few specimens of the rare Chrysomallon squamiferum snail were available to us. However, to minimise biases, sequencing and analyses of the data were performed and cross-checked by different research groups or researchers.

Did the study involve field work? ☒ Yes ☐ No

Field work, collection and transport

Field conditions	Deep-sea hydrothermal vent fields between 2415 m and 2606 m in depth, dives of the manned submersible Shinkai 6500 were carried out on days of calm sea condition. Local temperature where Chrysomallon squamiferum lived is between 8.4–13.5 degrees Celsius (average 12.6 degrees Celsius), dissolved oxygen between 133–187 µM (averaging 158 µM).
Location	Kairei field (25°19.23'S, 70°02.42'E, 2415 m depth) and Solitaire field (19°33.41'S, 65°50.89'E, 2606 m depth)
Access and import/export	Specimens of the Scaly-foot Snail (Chrysomallon squamiferum) used in the present study were collected by the manned submersible Shinkai 6500 on-board multiple deep-sea research expeditions of R/V Yokosuka. Sampling in the Mauritian EEZ was approved by Ministry of Foreign Affairs, Regional Integration, and International Trade, Mauritian Government (Permit Ref. 29/2014; 50/38/24 V2). All import/export were approved by the Mauritian and Japanese government under the same permit.
Disturbance	In order to minimise disturbance of the manned submersible Shinkai 6500 and sample collection to the natural hydrothermal vent environment, the submersible landed at least 150 m away from the vent field (which is only approx. 100 m by 100 m in size) and left the sea bottom only after confirming that the submersible has moved at least 100 m away from the edge of the vent field. During collection of animals, care was taken to not damage vent chimney structures or disrupt the animal colony more than the minimal necessary to do the collecting.

Reporting for specific materials, systems and methods

We require information from authors about some types of materials, experimental systems and methods used in many studies. Here, indicate whether each material, system or method listed is relevant to your study. If you are not sure if a list item applies to your research, read the appropriate section before selecting a response.

Materials & experimental systems

n/a	Involved in the study	
☒	☐	Antibodies
☒	☐	Eukaryotic cell lines
☒	☐	Palaeontology
☐	☒	Animals and other organisms
☒	☐	Human research participants
☒	☐	Clinical data

Methods

n/a	Involved in the study	
☒	☐	ChIP-seq
☒	☐	Flow cytometry
☒	☐	MRI-based neuroimaging

Animals and other organisms

Policy information about studies involving animals; ARRIVE guidelines recommended for reporting animal research

Laboratory animals	No laboratory animals were used in the study.
Wild animals	Scaly-foot Snails (Chrysomallon squamiferum) were collected in deep-sea hydrothermal vent by the manned submersible Shinkai 6500 on-board R/V Yokosuka. Collection was done using a slurp gun (suction sampler). Individuals used for gene expression analyses were killed and fixed in situ using RNAlater solution, in order to make sure the gene expression patterns analysed reflect those in the natural condition and not influenced by the changes in pressure, temperature, and other environmental factors during the submersible's ascend and recovery to the research vessel. Individuals used for genome/Hi-C sequencing were immediately placed in -80°C freezer when the submersible was recovered to the research vessel, in order to best preserve their intact genomic DNA.
Field-collected samples	Samples were either fixed in situ in the deep sea or immediately placed into -80°C freezer alive, upon recovery on the ship.
Ethics oversight	No ethical approval or guidance was required as the only species used (Chrysomallon squamiferum) is an invertebrate gastropod mollusc. Collections in the Mauritian EEZ was approved by Ministry of Foreign Affairs, Regional Integration, and International Trade, Mauritian Government (Permit Ref. 29/2014; 50/38/24 V2). All export of animal specimens were approved by the Mauritian and Japanese government under the same permit. Research cruises and their collecting activities were authorised and approved by the Japan Agency for Marine-Earth Science and Technology (numbers YK13-02, YK13-03, and YK16-E02).

Note that full information on the approval of the study protocol must also be provided in the manuscript.
